# Metabolic engineering with ATP-citrate lyase and nitrogen source supplementation improves itaconic acid production in *Aspergillus niger*

**DOI:** 10.1186/s13068-019-1577-6

**Published:** 2019-09-30

**Authors:** Abeer H. Hossain, Roy van Gerven, Karin M. Overkamp, Peter S. Lübeck, Hatice Taşpınar, Mustafa Türker, Peter J. Punt

**Affiliations:** 1 Dutch DNA Biotech B.V., Padualaan 8, 3584 CH Utrecht, The Netherlands; 2 Molecular Biology and Microbial Food Safety, Swammerdam Institute for Life Sciences, University of Amsterdam, Science Park 904, 1098 XH Amsterdam, The Netherlands; 3 Section for Sustainable Biotechnology, Department of Chemistry and Bioscience, Aalborg University, A.C. Meyers Vaenge 15, 2450 Copenhagen SV, Denmark; 4 Pakmaya, Kosekoy Mah. Ankara Cad. No:277, 41310 Kartepe, Kocaeli Turkey

**Keywords:** Itaconic acid, *Aspergillus niger*, Transcriptome analysis, Metabolic engineering, ATP-citrate lyase, Fermentation optimization

## Abstract

**Background:**

Bio-based production of organic acids promises to be an attractive alternative for the chemicals industry to substitute petrochemicals as building-block chemicals. In recent years, itaconic acid (IA, methylenesuccinic acid) has been established as a sustainable building-block chemical for the manufacture of various products such as synthetic resins, coatings, and biofuels. The natural IA producer *Aspergillus terreus* is currently used for industrial IA production; however, the filamentous fungus *Aspergillus niger* has been suggested to be a more suitable host for this purpose. In our previous report, we communicated the overexpression of a putative cytosolic citrate synthase *citB* in an *A. niger* strain carrying the full IA biosynthesis gene cluster from *A. terreus,* which resulted in the highest final titer reported for *A.* *niger* (26.2 g/L IA). In this research, we have attempted to improve this pathway by increasing the cytosolic acetyl-CoA pool. Additionally, we have also performed fermentation optimization by varying the nitrogen source and concentration.

**Results:**

To increase the cytosolic acetyl-CoA pool, we have overexpressed genes *acl1* and *acl2* that together encode for ATP-citrate lyase (ACL). Metabolic engineering of ACL resulted in improved IA production through an apparent increase in glycolytic flux. Strains that overexpress *acl12* show an increased yield, titer and productivity in comparison with parental strain CitB#99. Furthermore, IA fermentation conditions were improved by nitrogen supplementation, which resulted in alkalization of the medium and thereby reducing IA-induced weak-acid stress. In turn, the alkalizing effect of nitrogen supplementation enabled an elongated idiophase and allowed final titers up to 42.7 g/L to be reached at a productivity of 0.18 g/L/h and yield of 0.26 g/g in 10-L bioreactors.

**Conclusion:**

Ultimately, this study shows that metabolic engineering of ACL in our rewired IA biosynthesis pathway leads to improved IA production in *A. niger* due to an increase in glycolytic flux. Furthermore, IA fermentation conditions were improved by nitrogen supplementation that alleviates IA induced weak-acid stress and extends the idiophase.

## Introduction

A shortage of fossil fuels in the near future as well as their contribution to global carbon emission increases demand for renewable energy sources. Bio-based production promises to be an attractive alternative for the chemicals industry to substitute petrochemicals as building-block chemicals. In a landmark study in 2004, the US Department of Energy evaluated 300 molecules that could be made from biomass [1]. Twelve of these molecules were recognized as promising new building-block chemicals with high potential to be produced by biotechnological means, 8 of which belong to the organic acids. This list has been revisited by Bozell et al. [2] more recently. These biotechnologically produced organic acids are used, among others, in the food industry as food additive (e.g., citric acid) or in the chemicals industry as promising substitutes of petrochemicals (e.g., itaconic acid). In recent years, itaconic acid (IA, methylenesuccinic acid) has been established as a sustainable building-block chemical for the manufacture of various synthetic resins, coatings, and polymers and for application in thickeners, binders and adhesives [3–6]. In addition, IA has great potential to replace petroleum-based acrylic or methacrylic acid, used for synthesis of methyl methacrylate and its polymer polymethyl methacrylate (i.e., acrylic glass), and is suitable for synthesis of 3-methyltetrahydrofuran, a potential biofuel [4]. Its flexibility as a building-block chemical is expected to provide an increasing market for IA production.

The natural IA producer *Aspergillus terreus* is currently used for industrial IA production. Up to now, under laboratory conditions, this fungus has been shown to achieve a maximum productivity of 1.9 g/L/h with a final titer of 160 g/L IA in 1.5-L scale fermentations [7]. However, the conditions used in this study are not industrially scalable due to the use of specialized columns to remove trace amounts of Mn^2+^ from the medium, for which *A. terreus* is highly sensitive [8]. Industrial levels reported are in the range of 80–100 g/L IA [9]. IA can also be synthesized chemically, but this requires a multi-step modification process along with high substrate cost and relatively low yields and is, therefore, not preferred over bio-based production [3]. The world market of IA is around 40 kT per annum, with market prices between $1.5 and $2.0 per kg [10, 11].

Previously, the filamentous fungus *A. niger* has been suggested to be a suitable host for the industrial production of IA because of its optimized pathways towards organic acids, highlighted by its current status as key production host in industrial citric acid production with an estimated annual production exceeding 2 million tons (data from 2015) [12, 13]. Unlike *A. terreus*, *A. niger* is not able to produce IA and requires genetic modification to enable IA production. However, for several products, fermentation with *A.* *niger* has received the GRAS status (generally recognized as safe) from the US Food and Drug Administration, in contrast to *A.* *terreus* [14]. Another significant advantage of *A. niger* over *A. terreus* is its superior tolerance towards impurities that are abundantly present in industrial cultivation media [15, 16].

**Fig. 1:**
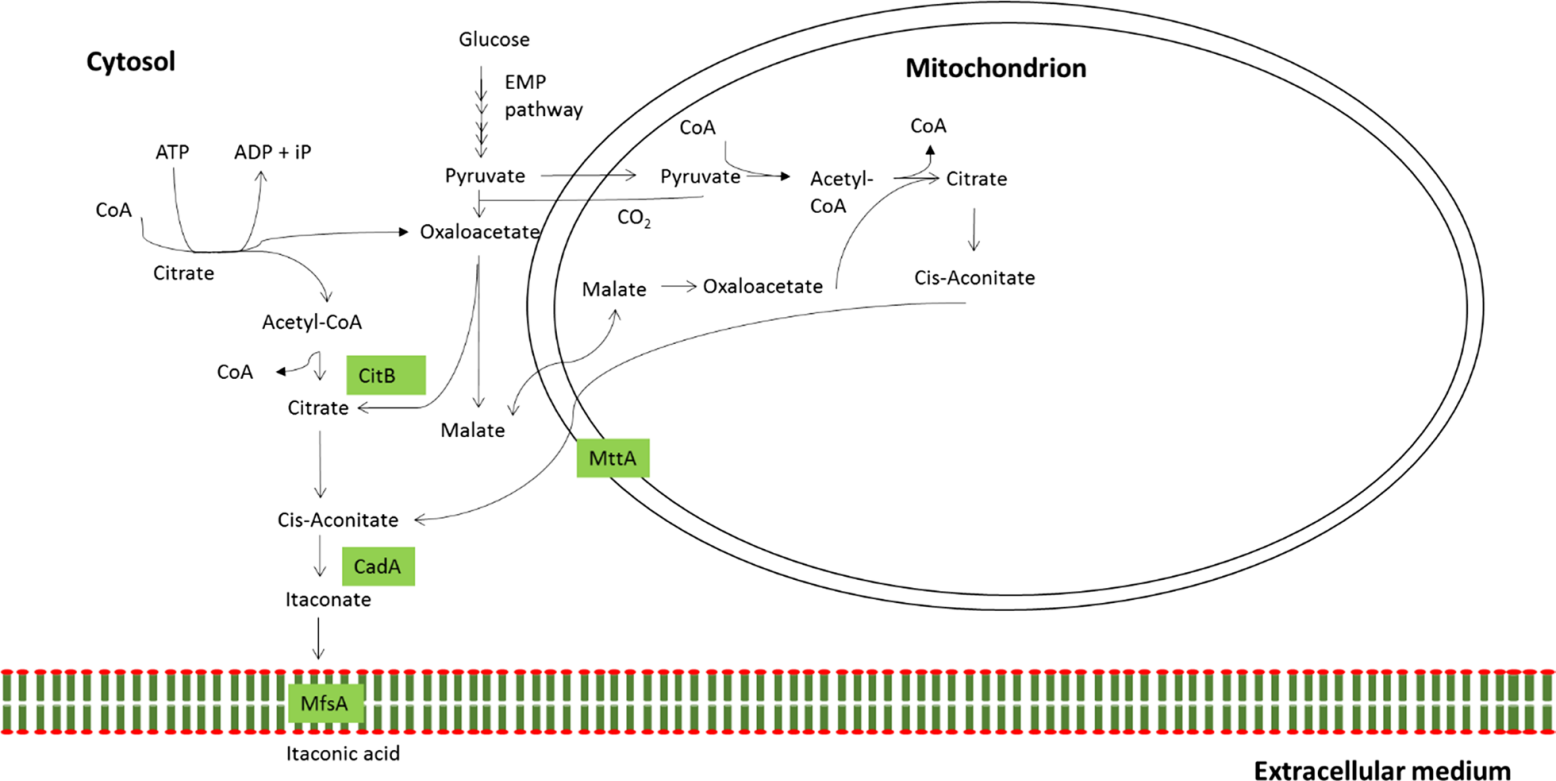
Proposed rewired IA biosynthesis pathway in *A. niger*. MttA (mitochondrial tricarboxylate transporter) transports the TCA-cycle intermediate *cis*-aconitate from the mitochondrion into the cytosol where CadA (*cis*-aconitate decarboxylase) decarboxylates *cis*-aconitate and thereby forms itaconate. Itaconate is transporter over the cellular membrane by action of MfsA (major facilitator superfamily protein). CitB (cytosolic citrate synthase) catalyzes the synthesis of citrate in the cytosol

In our previous report, we communicated the overexpression of a putative cytosolic citrate synthase *citB* in an *A. niger* strain carrying the full IA biosynthesis gene cluster from *A. terreus*, where we suggest that rewiring of CitB to heterologous IA production leads to the formation of a cytosolic IA biosynthetic pathway in which cytosolic acetyl-CoA and oxaloacetate are converted into citrate and ultimately converted into IA by CadA (Fig. 1) [17]. Remarkably, this resulted in the highest final titer reported for *A.* *niger* (26.2 g/L IA with a maximum productivity of 0.35 g/L/h in 5-L scale fermentations) [17]. We also hypothesized that cytosolic precursors for citrate synthesis (i.e., oxaloacetate and acetyl-CoA) might be a limiting factor in our rewired pathway, resulting in limited IA production. Whereas cytosolic oxaloacetate is mainly synthesized by the activity of pyruvate carboxylase, the synthesis and supply of cytosolic acetyl-CoA is more of an unknown factor [18, 19]. In the research presented here we have designed an approach to increase heterologous IA production rates by increasing cytosolic acetyl-CoA levels.

**Fig. 2:**
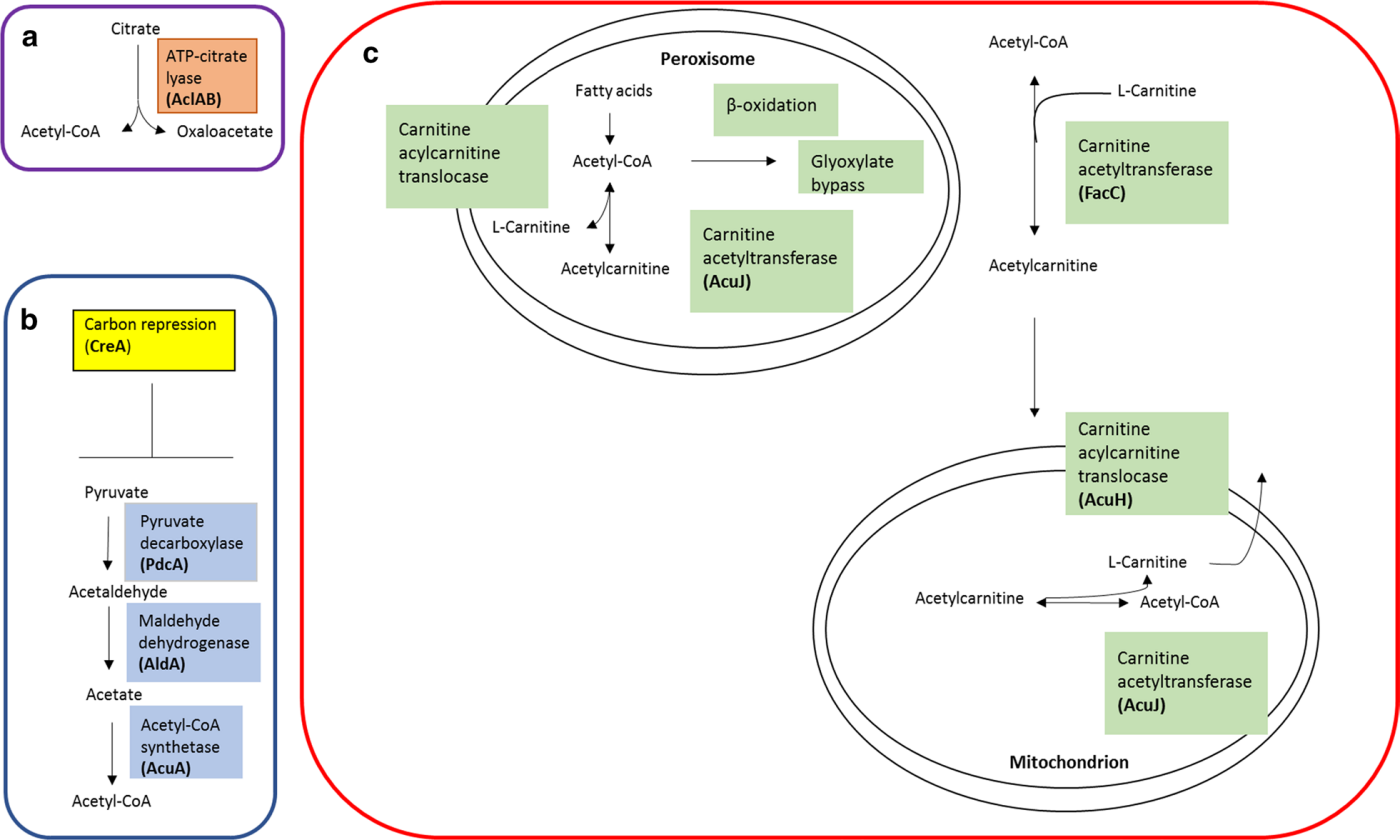
Cytosolic acetyl-CoA synthesis pathways described in literature. **a** ATP-citrate lyase (ACL) cleaves citrate into its constituent components oxaloacetate and acetyl-CoA at the expense of one ATP. **b** Pyruvate-Acetaldehyde-Acetate (PAA) pathway that converts pyruvate into acetyl-CoA by action of PdcA, AldA and AcuA. This pathway is directly repressed at the transcriptional level by CreA. **c** Carnitine acetyltransferase (CAT) system that consists of mitochondrial and peroxisomal carnitine acetyltransferase AcuJ or cytosolic FacC that catalyzes the reversible reaction of acetyl-CoA into acetylcarnitine. Acetylcarnitine in turn can be transported to and from the mitochondrion by carnitine acylcarnitine translocase (AcuH) or by a yet unknown translocase from the peroxisome

In literature, three pathways are described that can supply or synthesize cytosolic acetyl-CoA in filamentous fungi: through ATP-citrate lyase (ACL), the pyruvate–acetaldehyde–acetate pathway (PAA) and the carnitine acetyltransferase (CAT) system [18, 19] (Fig. 2). ACL has been described and characterized as an essential enzyme for the production of cytosolic acetyl-CoA in filamentous fungi and in particular *Aspergillus* [19, 20]. ACL (EC 2.3.3.8) consists of two different subunits which cleave citrate into its constituent components oxaloacetate and acetyl-CoA at the cost of one ATP (Fig. 2a). Increased activity of ACL was shown to increase the cytosolic pool of acetyl-CoA by Weyda et al. [21] in *Aspergillus carbonarius,* a species closely related to *A. niger*. In the yeast *Saccharomyces cerevisiae,* only the PAA pathway has been reported to be a significant source of cytoplasmic acetyl-CoA synthesis, while the ATP-citrate lyase route is completely absent [22]. In the PAA pathway, pyruvate is decarboxylated to acetaldehyde by action of pyruvate decarboxylase (PdcA) (EC 4.1.1.1). Acetaldehyde is acted upon by aldehyde dehydrogenase (ALD) (EC 1.2.1.3) to synthesize acetate which in turn is converted into acetyl-CoA by acetyl-CoA synthetase(ACS) (EC 6.2.1.1) (Fig. 2b). The PAA pathway may be an important pathway for cytosolic acetyl-CoA synthesis in *S. cerevisiae*; however, in *A. niger,* this pathway is an unlikely source of acetyl-CoA due to the low-level intracellular concentrations of the precursor acetate [23, 24]. Moreover, under glucose-grown conditions, the PAA pathway is repressed by the global carbon catabolite repressor CreA at the transcriptional level in the closely related fungal model organism *Aspergillus nidulans* [25]. The third possible pathway for cytosolic acetyl-CoA generation, the CAT system, consists of the mitochondrial and peroxisomal carnitine acetyltransferase AcuJ (EC 2.3.1.7) that catalyzes the reversible reaction of acetyl-CoA into acetylcarnitine. The cytosolic carnitine acetyltransferase FacC (EC 2.3.1.7) can catalyze the same reversible reaction in the cytosol. Together with the mitochondrial carrier protein AcuH, the CAT system provides a means to shuttle acetyl-CoA between the peroxisome, mitochondria and cytosol [18, 26, 27] (Fig. 2c). However, like the PAA pathway, the CAT system is not active under glucose grown conditions but only during growth on acetate or fatty acids [27].

Next to genetic engineering to improve the biosynthetic pathway, we have also focused our attention on optimizing the culture medium used for IA production. Current IA producing conditions were tailored to the first-generation low IA producing *A. niger* strains that were generated when *cadA* was first expressed in *A. niger* by Li et al. [28]. For this purpose, we have looked into literature to identify the IA producing conditions and medium components reported for *A. terreus* [8, 29]. Based on these reported conditions, we have designed a new IA production medium and fermentation protocol.

## Materials and methods

### Strains and culture conditions

Strains of *Aspergillus niger* used in this study have an AB1.13 background and are listed in Table 1 [30]. All strains were stored in 20% glycerol at − 80 °C and maintained on minimal medium (MM) agar [31]. Growth medium was supplemented with 10-mM uridine and 10-mM uracil when required. Spore suspensions were prepared by harvesting conidia from MM agar after 3–6 days of growth at 33 °C using 0.9% (w/v) NaCl and a sterile cotton stick. Spore suspensions were stored at 4 °C for up to 1 month without loss of viability.

**Table 1:** Strains of *Aspergillus niger* used in this study

Strain of *A. niger*	Abbreviation	Description
AB1.13 *pyrG*+	AB1.13	Uridine prototroph of AB1.13 *pyrG*- [32]
AB1.13 CAD 4.1	AB1.13 CAD	Selected pyrG + transformant of *cadA* expressing transformant (CAD10.1) of AB1.13 [28]
AB1.13 CAD + MFS + MTT + CitB #99	CitB#99	*citB* overexpressing strain of AB1.13 CAD + MFS + MTT #49B [17]
AB1.13 CAD + MFS + MTT + CitB #99-*acl* A4 R AB1.13 CAD + MFS + MTT + CitB #99-*acl* C3 R AB1.13 CAD + MFS + MTT + CitB #99-*acl* D9 R AB1.13 CAD + MFS + MTT + CitB #99-*acl* E11 Z AB1.13 CAD + MFS + MTT + CitB #99-*acl* F11 Z AB1.13 CAD + MFS + MTT + CitB #99-*acl* G1 Z AB1.13 CAD + MFS + MTT + CitB #99-*acl* G8 Z AB1.13 CAD + MFS + MTT + CitB #99-*acl* G11 Z AB1.13 CAD + MFS + MTT + CitB #99-*acl* H4 Z AB1.13 CAD + MFS + MTT + CitB #99-*acl* A9 G AB1.13 CAD + MFS + MTT + CitB #99-*acl* A12 G AB1.13 CAD + MFS + MTT + CitB #99-*acl* B11 G AB1.13 CAD + MFS + MTT + CitB #99-*acl* D10 G AB1.13 CAD + MFS + MTT + CitB #99-*acl* D12 G	ACL A4 R ACL C3 R ACL D9 R ACL E11 Z ACL F11 Z ACL G1 Z ACL G8 Z ACL G11 Z ACL H4 Z ACL A9 Z ACL A12 G ACL B11 G ACL D10 G ACL D12 G	*acl*1 and *acl*2 overexpressing strain of AB1.13 CAD + MFS + MTT + CitB #99 (this study)

### Molecular biological techniques

General cloning procedures in *E. coli* were according to Sambrook and Russell [33]. Restriction enzymes and buffers were obtained from Thermo Scientific or New England Biolabs and used according to the manufacturer’s instructions. DNA fragments were extracted from agarose gel using the QIAquick Gel Extraction Kit (QIAGEN) according to the manufacturer’s instructions. For amplification of plasmid DNA, chemically competent cells of *E. coli* strain DH5α (i.e., Thermo Scientific Subcloning Efficiency DH5α Competent Cells or Thermo Scientific Library Efficiency DH5α Competent Cells when required) were transformed by heat shock according to the manufacturer’s instructions. *E. coli* was grown in lysogeny broth (LB) at 37 °C. Plasmid DNA was isolated by miniprep using the GeneJET Plasmid Miniprep Kit (Thermo Scientific) or by maxiprep using the Plasmid Plus Maxi Kit (QIAGEN) according to the manufacturer’s instructions.

Fungal genomic DNA was isolated from mycelium cultivated in 1-mL liquid complete medium (CM) (MM + 2.5 g/L yeast extract) in 96-well microtiter plates, using the mag mini DNA extraction from µL blood kit (LGC) according to the manufacturer’s instructions. When high yield of genomic DNA was required, mycelium from 250-mL CM shake flask cultures was frozen in liquid nitrogen and ground to powder using a sterile mortar and pestle before extraction with the mag mini DNA extraction from µL blood kit. DNA concentration and purity were determined using the NanoDrop One^c^ UV–Vis Spectrophotometer (Thermo Scientific). PCR was performed on an Alpha Cycler 4 (PCRmax) using DreamTaq DNA Polymerase (Thermo Scientific) or Phusion Hot Start II DNA Polymerase (Thermo Scientific) when required. Amplified DNA from PCR was purified using the QIAquick PCR Purification Kit (QIAGEN) according to manufacturer’s instructions. Primers were obtained from Eurogentec and are listed in Table 2. DNA sequencing was carried out by BaseClear. Isolation of RNA, library preparation, sequencing and transcriptome data analysis are described in Hossain et al. [17].

**Table 2:** Primers used in this study

Primer name	F/R	Sequence 5′–3′	Template
332 AclA-3,600 F	F	TCAGCCCAGGTCT-TGTTCAG	*A. carbonarius acl2* ORF
333 AclA-4,237 R	R	CCCTCCATGTCCA-ATGATAAGGA	*A. carbonarius tef1* promoter
334 AclB-3,910 F	F	TCCCTTCCCTCGCT-TCTCTC	*A. nidulans tef1* promoter
335 AclB-4,497 R	R	ATGGCCTTGCTGA-CATCCTG	*A. carbonarius acl1* ORF

### Cloning, transformation and cultivation of *A. niger*

The *acl* overexpression vectors pSBi415aACLoe and pSBi415bACLoe, containing the *acl1* and *acl2* expression cassettes as designed by Weyda et al. [21], were introduced into *E. coli* for amplification of plasmid DNA (Additional file 1: Figure S1).

Auxotrophic *pyrE*-mutants of CitB#99 were generated by cultivation on selective MM plates containing 5-fluoroorotic acid for 3–5 days at 33 °C until colonies appeared [34]. Organic acid production of CitB#99 *pyrE*-mutants was examined by cultivation in a 1-mL liquid culture of IA production medium M12++ (1.43 g/L NH_4_NO_3_, 0.11 g/L KH_2_PO_4_, 0.5 g/L MgSO_4_ × 7H_2_O, 0.005 g/L CuSO_4_ × 5H_2_O, 0.0006 g/L FeCl_3_ × 6H_2_O, 0.0006 g/L ZnSO_4_ × 7H_2_O, 0.074 g/L NaCl, 0.13 g/L CaCl_2_ × 2H_2_O, 100 g/L glucose, set at pH 2.3 with 10 M H_2_SO_4_), adapted from Li et al. [28]) in a 96-well deep-well plate that was incubated for 5 days at 33 °C, 850 rpm. To create an *acl12* overexpression strain of CitB#99, one of the generated CitB#99 *pyrE*-mutants was transformed with both pSBi415aACLoe and pSBi415bACLoe simultaneously, by co-transformation with pJET1.2-*pyrE*, harbouring the *pyrE* auxotrophic marker (i.e., gene encoding orotate P-ribosyl transferase) from *Aspergillus oryzae,* in a ratio of 1:10 (5 µg of each construct:0.5 µg marker). Fungal transformation procedures were according to Arentshorst et al. [35]. Transformants were selected for their ability to grow on MM agar containing 115 g/L sucrose or 109.3 g/L sorbitol without uridine/uracil (i.e., uracil prototrophy).

### Transformant screening

Individual colonies from transformation plates were transferred to a 48-well plate containing selective MM agar using a sterile toothpick that in turn was used to inoculate a 1-mL liquid culture of complete medium (CM), consisting of MM with the addition of 0.5% (w/v) yeast extract and 0.1% (w/v) casamino acids, or M12++ medium in a 96-well deep-well plate that was incubated for 3–5 days at 33 °C, 850 rpm. Supernatant was removed when mycelia were grown sufficiently, followed by isolation of genomic DNA as described in “Molecular biological techniques”. Isolated genomic DNA was used as template in diagnostic PCR to screen for transformants containing the constructs of interest, using primers 332 + 333 for pSBi415aACLoe and primers 334 + 335 for pSBi415bACLoe. Positive transformants were streaked from the 48-well plate on selective MM agar using 0.9% (w/v) NaCl and a sterile cotton stick to obtain pure single colony transformants, which were used to prepare spore suspensions as described in ‘Strains and culture conditions’.

### Shake flask cultivation and related analyses

For analysis of organic acid production and glucose consumption of *acl12* positive transformants, shake flasks (500 mL) containing 100 mL M12++ medium or MRA medium (3 g/L NH_4_NO_3_, 0.11 g/L KH_2_PO_4_, 1 g/L MgSO_4_ × 7H_2_O, 0.005 g/L CuSO_4_ × 5H_2_O, 0.0016 g/L FeCl_3_ × 6H_2_O, 0.0006 g/L ZnSO_4_ × 7H_2_O, 0.074 g/L NaCl, 0.13 g/L CaCl_2_ × 2H_2_O, 100 g/L glucose, set at pH 2.3 with 10 M H_2_SO_4_) were inoculated with 1.0 × 10^6^ spores/mL and cultivated for up to 2 weeks at 33 °C, 250 rpm. A 200-µL sample was filtered over a 0.22-µm filter daily to determine organic acid and glucose concentrations in the extracellular medium using High-Performance Liquid Chromatography (HPLC). Cultures were weighed before sampling to account for evaporation of the culture medium. Shake flask cultures were supplemented with 17.87 mM NH_4_NO_3_, 35.74 mM NH_4_Cl or 35.74 mM NaNO_3_ before titers reached 20 g/L IA during experiments on nitrogen supplementation. Supplementation continued beyond this titer at time intervals of 2–3 days when nitrogen was estimated to be depleted. Equimolar amounts of nitrogen were supplemented at once for all nitrogen sources.

Analysis of metabolite production was performed by HPLC using a WATERS e2695 separations module equipped with an Aminex HPX-87H column (Bio-Rad) and 5-mM H_2_SO_4_ as eluent. Column temperature was set at 60 °C and eluent flow was 600 µL/min. Peak detection occurred simultaneously by a refractive index detector (WATERS 2414) and a dual-wavelength detector (WATERS UV/Vis 2489). Empower Pro software (Empower 2 Software, copyright 2005–2008, Waters Corporation) was used for data processing.

### Southern blotting

For Southern blotting of CitB#99-*acl* transformants, shake flask cultures containing 100-mL CM (pH 4.5) were inoculated with 1.0 × 10^5^ spores/mL and grown for 2 days at 33 °C, 200 rpm. Mycelia were harvested using a sterile Miracloth filter, dried using paper towels and frozen in liquid nitrogen. Genomic DNA was isolated from 300-µg ground mycelium per strain as described in “Molecular biologic techniques”. Quality of isolated DNA was tested by running on 0.8% (w/v) agarose gel. 1-µg genomic DNA was digested per strain using *Pvu*II and run on agarose gel O/N. Blotting occurred on nitrocellulose Hybond-N + blotting paper (Amersham Biosciences) using SSC solution (175.5-g NaCl and 88.2-g Na-Citrate in 1 L; 20 × stock). After blotting, the membranes were treated in a UV-chamber to cross-link DNA. Digoxigenin-labeled (DIG) probes for *acl*1 and *acl*2 were created by PCR from pSBi415bACLoe using primers 334 + 335 and from pSBi415aACLoe using primers 332 + 333, respectively, using the PCR DIG Probe Synthesis Kit (Roche Life Sciences) according to the manufacturer’s instructions. Labelling and detection were performed using the DIG Easy Hyb labelling and detection kit for Southern blotting purposes (Roche Life Sciences) according to the manufacturer’s instructions. The ChemiDoc XRS + Imaging System (Bio-Rad) was used for imaging of Southern blots.

### Controlled fed-batch cultivations and related analyses

Controlled fed-batch cultivations in 16-L stainless steel stirred tank bioreactors (NLF22, Bioengineering, Switzerland) were conducted at 33 °C with a working volume of 10 L. Cultivation occurred in M2 medium that consisted of 2.29 g/L NH_4_NO_3_, 0.18 g/L KH_2_PO_4_, 0.80 g/L MgSO_4_ × 7H_2_O, 0.008 g/L CuSO_4_ × 5H_2_O, 0.001 g/L FeCl_3_ × 6H_2_O, 0.001 g/L ZnSO_4_ × 7H_2_O, 0.118 g/L NaCl, 0.21 g/L CaCl_2_ × 2H_2_O and 160 g/L glucose. Dissolved oxygen levels were maintained above 30% by controlling the stirrer speed and aeration rate between 450 and 800 rpm and 3–8 L/min, respectively. The pH was maintained at 3.0 after 48-h EFT by continuous addition of 3-M NH_4_OH. Additionally, the pH was controlled offline twice per day to exclude errors of the online sensor. Bioreactor inoculum was prepared by filling a 1-L non-baffled shake flask with 200-mL M12++ medium and inoculating with 1.0*10^6^ spores/mL and cultivated for 96 h at 33 °C and 250 RPM. A sterile 700 g/L glucose solution was used for addition of sugar during runs. For determination of the cell dry weight, biomass was separated from fermentation broth using vacuum filtration and washed with deionized water, then dried at 105 °C for 48 h. The exhaust gas of the fermentation was analyzed with a BioPAT Xgas analyzer (Sartorius Stedim Biotech GmbH, powered by BlueSens) on %CO_2_ and %O_2_.

For quantification of sugars, organic acids and nitrogen in the media, samples were filtered through a 0.45-µm filter (Minisart RC, Sartorius, Germany) and analyzed by HPLC (1100 Series, Agilent Technologies, USA). Total nitrogen and ammonia concentrations were determined by Kjeldahl and distillation–titration methods, respectively [36]. Phosphate content was analyzed by a colorimetric method using LCK 349 Phosphate Kit (HACH).

## Results

### Analysis of cytosolic acetyl-CoA generating pathways

Rewiring CitB to heterologous IA production resulted in increased production titers (26.2 g/L IA). From our previous research, it was hypothesized that acetyl-CoA might be a limiting factor for cytosolic IA production in the rewired pathway [17]. Therefore, transcriptome analysis of possible acetyl-CoA pathways was carried out in the AB1.13 WT strain and AB1.13 CAD strain to understand which pathways predominantly generate cytosolic acetyl-CoA in *A. niger.* As described above from literature, it was discerned that three pathways are identified as cytosolic acetyl-CoA generating pathways in fungi: through ACL that cleaves cytosolic citrate into oxaloacetate and acetyl-CoA, the PAA pathway that converts pyruvate into acetyl-CoA and the CAT system that shuttles acetyl-CoA in the form of acetylcarnitine from and between the peroxisome and mitochondrion into the cytosol (Fig. 2) [18, 19, 37]. Interestingly, our transcriptome analysis shows that the expression of PAA pathway gene *pdcA* is strongly downregulated in the IA producing AB1.13 CAD strain compared to its parental strain AB1.13 (Table 3). It has been shown in a study conducted by Meijer et al. [38] that the PdcA enzyme was inactive in oxygen-limiting conditions. The reason for the low expression of *pdcA* in AB1.13 CAD and its link with heterologous IA production is, however, at the moment, not clear. Furthermore, from our transcriptome data (Table 3), we also see that the ACS coding gene (*acuA*) is expressed at low levels, in accordance with findings that this gene is under control of carbon catabolite repression [39, 40].

**Table 3:** Transcriptome data of known cytosolic acetyl-CoA generating pathways and the genes related to heterologous IA production in *A. niger*

Locus tag	Enzyme	Old locus tag	Localization	AB1.13 WT		AB1.13 CAD	
				RPKM		RPKM	
	ATP-citrate lyase						
ANI_1_76094	ATP-citrate lyase subunit 1 (*aclA*)	An11g00510	Cyto	325.61	325.45	506.32	505.54
ANI_1_78094	ATP-citrate lyase subunit 2 (*aclB*)	An11g00530	Cyto	331.2	332.23	587.4	587.02
	CAT system						
ANI_1_724074	Carnitine acetyl transferase (*facC*)	An08g04990	Cyto/Nucleus	28.78	29.17	24.89	24.77
ANI-1-192164	Carnitine acetyl transferase (*acuJ*)	An18g01590	Per/Mito	45.97	47.7	38.2	38.08
ANI_1_388034	Carnitine/acyl carnitine carrier (*acuH*)	An03g03360	Cyto/Mito	31.08	30.93	29.01	28.95
	PAA pathway						
ANI_1_936024	Pyruvate decarboxylase (*pdcA*)	An02g06820	Cyto	5910.75	5915.54	304.97	306.49
ANI_1_1024084	Pyruvate decarboxylase (*pdcB*)	An09g01030	Cyto	38.82	38.64	47.95	48.26
ANI_1_2276014	Pyruvate decarboxylase	An01g01590	Nucleus	0.35	0.45	0.17	0.18
ANI_1_796114	Pyruvate decarboxylase	An13g03320	Mito/Cyto	0	0	0.03	0.03
ANI_1_1024074	Aldehyde dehydrogenase (*aldA*)	An08g07290	Cyto	143.48	142.19	178.07	177.72
ANI_1_226174	Aldehyde dehydrogenase	An10g00850	Per	1.59	1.35	1.69	2.21
ANI_1_1748184	Aldehyde dehydrogenase	An04g03400	Cyto	54.25	54.85	53.22	54.15
ANI_1_924184	Acetyl-CoA synthetase (*acuA*)	An04g05620	Cyto	139.46	141.21	96.66	96.09
ANI_1_938144	Acetyl-CoA hydrolase (*ach1*)	An16g07110	Mito	98.53	99.38	22.36	22.93
ANI_1_878024	Acetate kinase	An02g06420	Cyto/Mito	42.53	43.41	13.72	13.75
	Mitochondrial acetyl-CoA synthesis						
ANI_1_1206064	Pyruvate dehydrogenase E1 component subunit alpha (*pda1*)	An07g09530	Mito	263.81	264.72	205.40	207.09
ANI_1_622094	Pyruvate dehydrogenase E1 component subunit alpha	An11g04550	Mito	10.81	9.47	10.54	9.75
ANI_1_12014	Pyruvate dehydrogenase E1 component subunit beta	An01g00100	Mito	215.18	213.19	177.40	176.43
ANI_1_274064	Pyruvate dehydrogenase E2 component	An07g02180	Mito	299.81	301.04	286.65	284.54
	Citrate synthases						
ANI_1_876084	Citrate synthase (*citA*)	An09g06680	Mito	478.40	482.85	428.47	426.67
ANI_1_1226134	Methylcitrate synthase (*mcsA*)	An15g01920	Mito	51.49	51.55	25.69	24.11
ANI_1_1474074	Citrate synthase (*citB*)	An08g10920	Cyto	58.09	57.90	522.44	521.45
ANI_1_2950014	Citrate synthase (*citC*)	An01g09940	Cyto	3.86	3.52	0.91	0.96
	Aconitases						
ANI_1_1410074	Aconitate hydratase (*aco1*)	An08g10530	Mito	234.09	234.83	397.94	397.68
ANI_1_470084	Aconitate hydratase	An09g03870	Mito	64.88	64.41	26.01	26.07
ANI_1_3018024	Aconitate hydratase	An02g11040	Cyto	0.04	0.04	0.00	0.00
ANI_1_1808144	Aconitate hydratase	An16g05760	Cyto	0.85	0.85	0.98	0.99
ANI_1_578044	Aconitase	An05g02230	Cyto	6.29	6.38	14.13	14.28
ANI_1_1802134	Aconitase	An15g07730	Cyto	26.63	26.21	30.62	30.72
	Itaconate biosynthesis						
	*cis*-aconitate decarboxylase (*cadA*)		Cyto	1.27	1.20	4287.14	4300.13

Gene expression data as obtained from Hossain et al. [17] are given in RPKM (Reads assigned Per Kilobase of target per Million mapped reads) values and calculated according to the method presented by Mortazavi et al. [41]

The picture that derives from transcriptome analysis indicates that the ACL pathway may actually be the major cytosolic acetyl-CoA generating pathway in *A. niger* (Table 3). This observation is also in accordance with the observations from Pfitzner et al. [24]. They have previously shown that ACL is ubiquitously present in *A. niger* cell lysates independent of the C-sources tested. Moreover, acetate, an intermediate in the PAA pathway, was below detection limit in cellular extracts; whereas, citrate was always present in detectable amounts [24]. These results are in accordance with our observation of significant expression of *acl1* and *acl2.* Therefore, and together with the results presented by Weyda et al. [21], we decided to overexpress *acl1* and *acl2* for increased cytosolic acetyl-CoA generation.

### Overexpression of *acl12*

To enhance IA production beyond titers reached with CitB#99, overexpression of *acl12* (i.e., overexpression of the *A. carbonarius* orthologues of the *A. niger* ACL subunit encoding genes *aclA* and *aclB*) was established in this strain. Presence of both *acl1* and *acl2* overexpression vectors in the genomes of 14 out of 144 transformants was confirmed by diagnostic PCR (data not shown). HPLC analysis of shake flask cultivations with *acl12* overexpressing CitB#99 transformants and the parental strain CitB#99 in M12++ medium with 125 g/L glucose showed that 9 CitB#99-*acl* transformants had an increase in max. IA titer compared to CitB#99, but most apparent was the increase in max. productivity of all transformants, excluding two strains with impaired IA production (i.e., ACL F11 Z and A12 G) (Table 4). In none of the strains, significant levels of other organic acids were detected. These results, particularly the increase in max. IA productivity, are in accordance with the expected increase in glycolytic flux upon overexpression of the ACL encoding genes. ACL G1 Z showed the highest increase (32%) in max. titer (20.6 g/L IA) of all CitB#99-*acl* transformants compared to the parental strain (15.6 g/L IA) (Table 4). This transformant outperformed all other transformants in overall IA production, also given its max. productivity of 0.20 g/L/h and yield of 0.30 g/g compared to the max. productivity and yield of the parental strain CitB#99 (0.13 g/L/h and 0.23 g/g) (Table 4).

**Table 4:** Itaconic acid production of *acl12* overexpressing CitB#99 transformants

Strain	Max. titer (g/L)	Max. productivity (g/L/h)	Yield (g/g glucose)
CitB#99	15.6	0.13	0.23
CitB #99 ACL A12 G	2.2	0.02	0.04
CitB #99 ACL F11 Z	10.5	0.11	0.15
CitB #99 ACL D12 G	14.2	0.14	0.22
CitB #99 ACL A4 R	15.2	0.14	0.23
CitB #99 ACL D10 G	15.3	0.14	0.23
CitB #99 ACL H4 Z	15.7	0.15	0.24
CitB #99 ACL G11 Z	16.2	0.15	0.24
CitB #99 ACL G8 Z	16.30	0.16	0.25
CitB #99 ACL C3 R	16.6	0.16	0.22
CitB #99 ACL A9 G	17.9	0.18	0.25
CitB #99 ACL B11 G	18.0	0.17	0.26
CitB #99 ACL E11 Z	18.4	0.17	0.26
CitB #99 ACL D9 R	18.4	0.17	0.28
CitB #99 ACL G1 Z	20.6	0.20	0.30

Results are from a single representative shake flask experiment for each transformant

Notably, ACL G1 Z reached a max. titer of 24.8 g/L IA in a similar shake flask cultivation, along with a max. productivity of 0.28 g/L/h and a yield of 0.33 g/g (Additional file 1: Figure S2). Southern blot analysis of the five best IA producing CitB#99-*acl* transformants and parental strain CitB#99 showed that ACL G1 Z did not possess a 1:1 ratio of *acl1* and *acl2*, harboring one copy of *acl1* and three copies of *acl2* (Additional file 1: Table S1). Overall, no correlation was found between IA production and number and/or ratio of gene copies of *acl1* and *acl2* present in the genomes of the CitB#99-*acl* transformants that were analyzed by Southern blotting (Table 4; Additional file 1: Table S1). Results in Additional file 1: Table S1 are derived from Southern blots shown in Additional file 1: Figure S3.

### Culture optimization and alkalizing nitrogen source supplementation

**Fig. 3:**
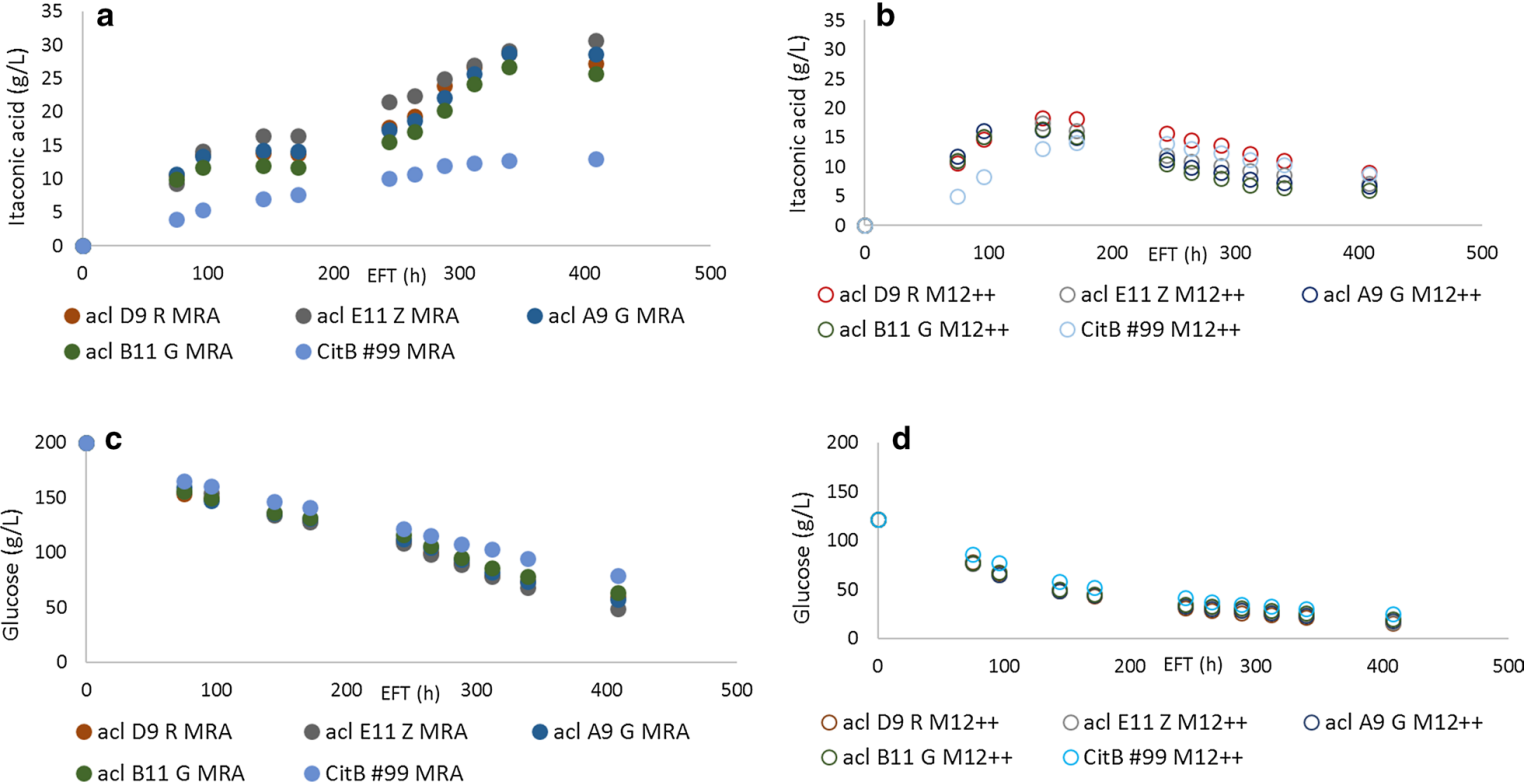
IA production of CitB#99 and CitB#99-*acl* overexpressing strains. **a** IA production in MRA medium. **b** IA production in M12++ medium. **c** Glucose consumption in MRA medium. **d** Glucose consumption in M12++ medium. Results depicted are from a single representative shake flask experiment for each transformant

Having established a new generation of improved IA producing *A. niger* strains, we have subsequently focused our attention on optimizing the culture medium used for IA production. Adjustments to the M12++ medium previously developed included an increase in glucose, nitrogen, magnesium and iron (III) concentration, based on a published IA production medium used for *A. terreus* [8, 29]. The resulting medium was termed MRA and its exact composition is described in the Materials and Methods. HPLC analysis of shake flask cultivations with *acl* transformants ACL D9 R, ACL E11 Z, ACL A9 G and ACL B11 G showed that these transformants reached titers ranging from 26.7 to 30.6 g/L IA when cultivated in MRA medium with 200 g/L glucose (Fig. 3). The average increase in max. titer was 58% compared to cultivation in M12++ medium with 125 g/L glucose. Although max. titers improved significantly upon cultivation in MRA medium compared to M12++ medium, both overall yield and max. productivity decreased under these culture conditions with average decreases of 16% and 13%, respectively. Note that although less glucose was provided here during cultivations in M12++ medium, this does not explain the lower titers reached given that glucose was not yet depleted when IA production stalled. Interestingly, it appears that in MRA medium, IA production shows two stages of production with an intermediary lag-phase in between the two production phases. From inoculation up to 96-h EFT, IA was being produced at a constant rate of 0.14 ± 0.05 g/L/h in MRA medium and 0.15 ± 0.04 g/L/h in M12++ medium. Between 96-h and 168-h EFT, IA production appears to have reached a plateau and no production appears to occur in both media. However, after 244-h EFT, IA production commenced further in MRA medium but not in M12++ medium, where a clear decline in IA titer can be observed. We have observed in the past that IA titers beyond 20 g/L can have a toxic effect on mycelia and thereby severely limit growth and biomass formation [17]. The observation that additional NH_4_NO_3,_ among others, was able to induce IA production beyond titers that are normally toxic and inhibitory for *A. niger* is interesting and was further investigated by feeding different nitrogen sources during shake flask cultivation.

To determine whether a specific nitrogen source was responsible for this effect, shake flask cultures with M12++ medium were supplemented with either NH_4_NO_3_, NaNO_3_ or NH_4_Cl upon reaching inhibitory conditions at titers of 15–20 g/L IA. Pulse feed was chosen over an increased initial nitrogen concentration in the culture medium to prevent any inhibition of IA production by excess nitrogen availability [4, 42].

**Fig. 4:**
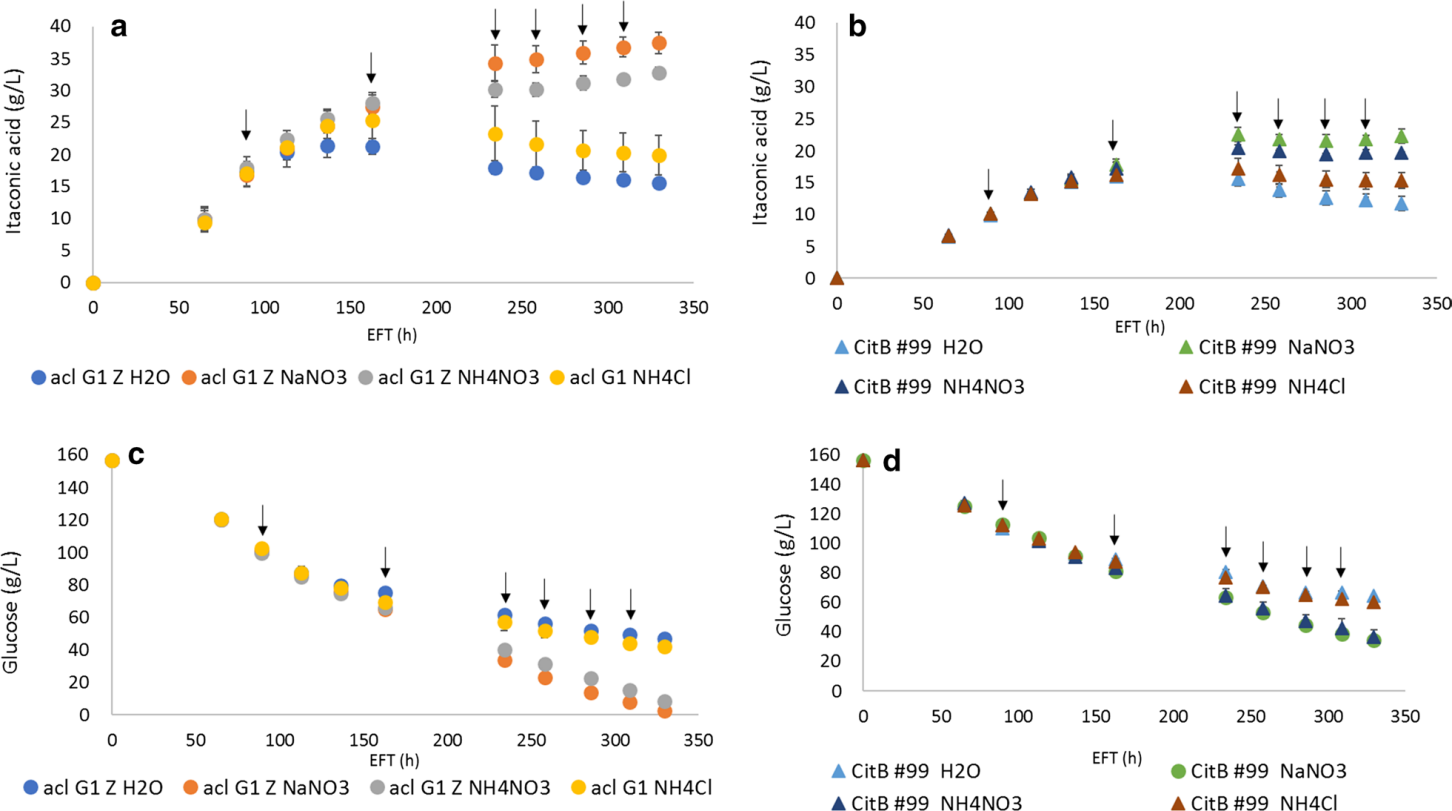
Nitrogen supplementation of CitB#99 and ACL G1 Z during cultivation in M12++ medium. Cultivation flasks inoculated with strains CitB#99 and ACL G1 Z were pulse fed with dH_2_O, NaNO_3_, NH_4_NO_3_ or NH_4_Cl (pulse feed indicated by arrow). **a** IA production of ACL G1 Z. **b** IA production of CitB#99. **c** Glucose consumption of ACL G1 Z. **d** Glucose consumption of CitB#99. Experiment was performed in duplicate. Error bars represent SEM (Standard Error of the Mean). Note that in those cases where the error bar is not visible it falls within the size of the datapoints

HPLC analysis results of nitrogen fed shake flask cultivations of ACL G1 Z and its parental strain CitB#99 are shown in Fig. 4. Interestingly, it was found that strains supplemented with NH_4_NO_3_ or NaNO_3_ extended their production phase till glucose was fully consumed, while strains not supplemented (dH_2_O) or supplemented with NH_4_Cl stopped producing at titers around 20 g/L IA (Fig. 4), although pulse feed with NH_4_Cl caused a slight increase in IA production compared to pulse feed with dH_2_O. This effect was observed for both strains ACL G1 Z and CitB#99. ACL G1 Z reached an average max. titer of 25.3 g/L IA when the culture medium was repeatedly supplemented with NH_4_Cl, after which IA titers went into decline, while IA production still continued to titers of 32.8 and 37.5 g/L IA when the culture medium was repeatedly supplemented with NH_4_NO_3_ or NaNO_3_, respectively (Fig. 4). These results show that availability of NO_3_^−^ and to a lesser degree NH_4_^+^ is important for *A.* *niger* to cope with toxic titers of IA, being able to extend the production phase beyond inhibitory conditions. Note that even though nitrogen sources were pulse fed, productivity decreased upon increasing IA titers suggesting depletion of other limiting compounds. These findings were taken into account during further research of ACL G1 Z under controlled fermentation conditions.

### Controlled fed-batch cultivations

**Fig. 5:**
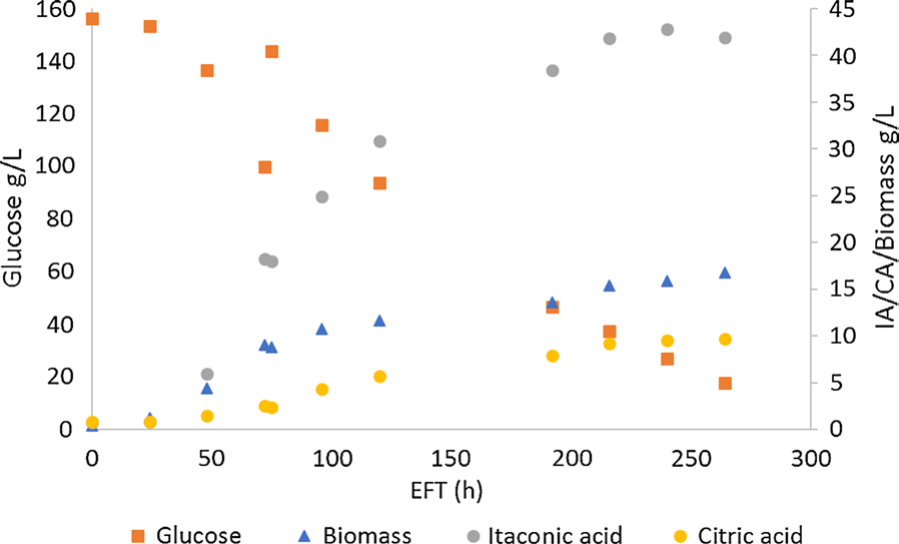
Controlled fed-batch cultivation of ACL G1 Z. IA production, CA production, biomass formation and glucose consumption is depicted. Performed as single experiment

In nitrogen fed shake flask experiments we have observed higher IA production when compared to non-fed flasks, as shown above. In particular, shake flasks fed with a N-source that has an alkalizing effect on the medium (i.e., NaNO_3_ and NH_4_NO_3_), had a positive effect on IA titer (Fig. 4). To further test IA production of ACL G1 Z, this strain was cultivated in controlled 10-L fed-batch fermentations where the pH was maintained at 3.0 using 3-M NH_4_OH. IA production, biomass formation and sugar consumption are shown in Fig. 5. Glucose concentration rapidly dropped from 160 to 100 g/L after which glucose was pulse fed. IA concentration at moment of feed was 18 g/L and biomass dry weight was 10 g/L. IA production further continued to a max. titer of 42.7 g/L after 240-h EFT, after which IA titer did not increase further. The overall productivity and yield at which IA was produced in this experiment was 0.18 g/L/h and 0.26 g IA/g glucose, respectively. During the course of fermentation, some citric acid formation was observed (max. titer 9.7 g/L; Fig. 5). The carbon balance accounted for 89.9% of total carbon that was added to the fermentor (Additional file 1: Table S2). The pH was steadily maintained at 3.0 after 48-h EFT by addition of 3-M NH_4_OH (Additional file 1: Figure S4). Although we attempted to keep the DO at minimal 30% by manually adjusting the airflow, at some points during the cultivation, the DO dropped below 30% (Additional file 1: Figure S4).

Biomass increased from 10 g/L after 60 h EFT to 16 g/L at the end of fermentation (264-h EFT). Nitrogen concentration in the media decreased in the first 48 h in accordance with the cell growth phase; however, after 48-h EFT nitrogen accumulation occurred, due to pH control with NH_4_OH addition (Additional file 1: Figure S5).

## Discussion

This research aimed to improve the biological production of IA by targeting its heterologous biosynthesis in *A. niger*. IA biosynthesis could be improved by overexpression of the ATP-citrate lyase encoding genes *acl1* and *acl2*. Furthermore, alkalizing nitrogen source availability turned out to be crucial for achieving high IA titers, which were greatly improved by the use of an IA production medium with increased nitrogen concentration or by supplementation of the culture medium with alkalizing nitrogen sources, i.e., nitrate and ammonia, extending the IA production phase.

**Fig. 6:**
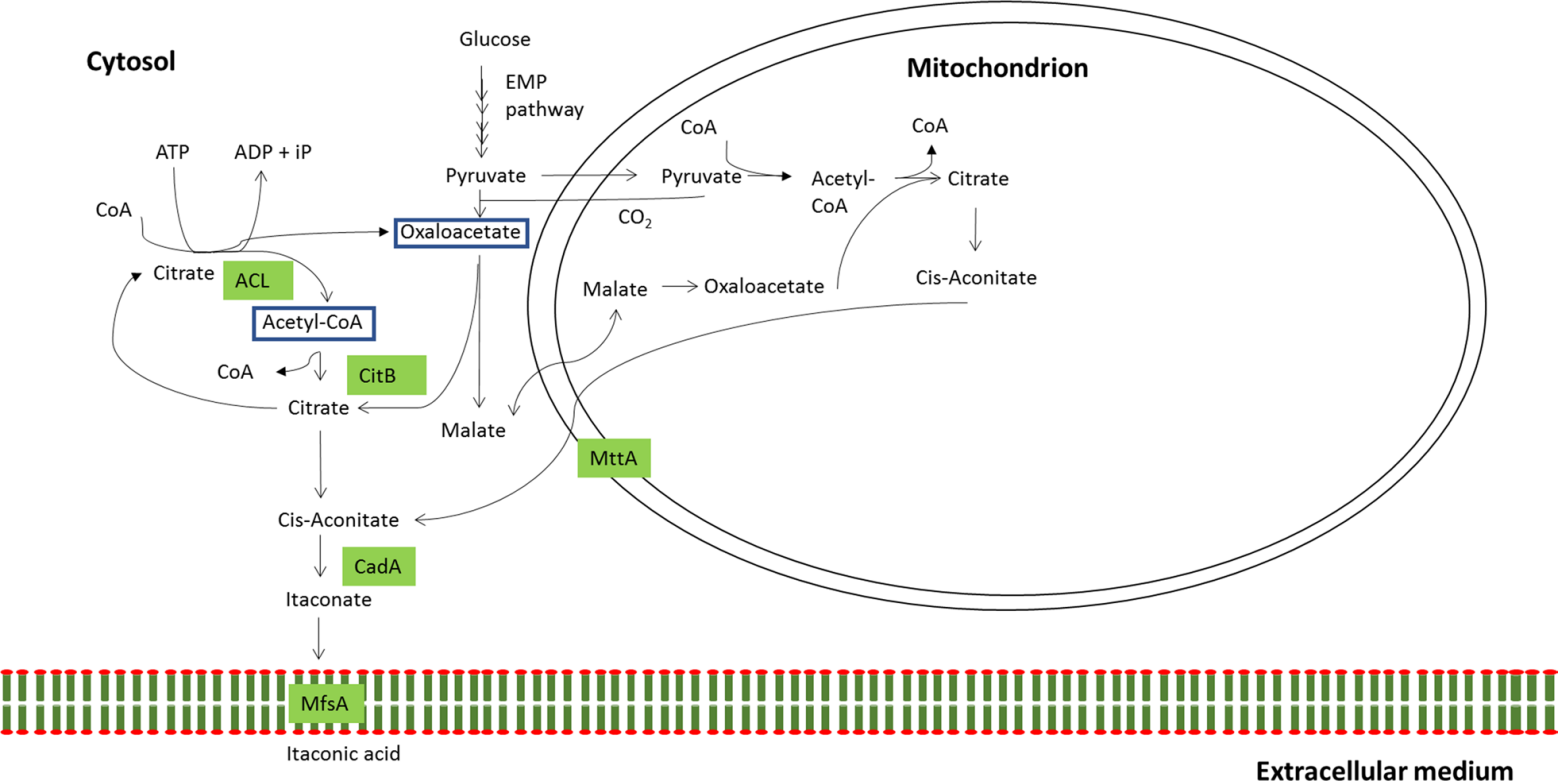
Proposed rewired IA biosynthesis pathway in *A. niger* that contains a futile cycle of citrate synthesis and cleavage through action of CitB and ACL, respectively. See also Fig. 1

The products of ACL are direct precursors for the generation of citrate by CitB and would, therefore, seemingly benefit the flux towards IA biosynthesis by facilitating the CitB precursor pool. However, it should be noted that the combined reactions of ACL and CitB constitute a futile cycle in which citrate is both substrate and product (Fig. 6). We interpret the increase in IA max. titer and max. productivity as a function of an increase in glycolytic flux that results from ATP consumption during the catabolic activity of ACL, which is supported by Wellen et al. [43] who showed that silencing the ACL encoding genes in mammalian cells leads to a significant decrease in cellular glycolytic activity. Additionally, Yin et al. [44] have shown that an ACL-mediated futile cycle of citrate cleavage and synthesis during idiophase is of importance to achieve high-yield citric acid production in *A. niger*. The increase in glycolytic flux ultimately benefits the flux towards IA biosynthesis by increasing precursor formation via the conversion of glucose to pyruvate and finally to oxaloacetate by pyruvate carboxylase in the cytosol and/or synthesis of *cis*-aconitate in the TCA cycle. This could arguably prevent the flux of potential IA precursors towards metabolic pathways other than for IA biosynthesis, explaining the observed increase in yield of the better IA producing CitB#99-*acl* transformants (Table 4).

A correlation between IA production and gene copy number of *acl1* and *acl2* in the genomes of the CitB#99-*acl* transformants was not found (Table 4, Additional file 1: Table S1). Because both genes encode one subunit of the ACL enzyme, the optimal ratio was expected to be 1:1 for *acl1* and *acl2*. The best IA producing CitB#99-*acl* transformant (i.e., ACL G1 Z) was not necessarily the closest one to this ratio compared to the other transformants analyzed by Southern blotting. However, this does not exclude the possibility that, of all *acl12* overexpression transformants, this strain is the closest to a 1:1 ratio in terms of expression of both genes, since besides number of gene copies, gene expression is also highly dependent on their site of integration in the genome.

Besides linking carbon metabolism to fatty acid synthesis, it was shown by Wellen et al. [43] that ACL also plays a central role in protein acetylation, including histone acetylation in the nucleus. In detail, these researchers found that ACL-dependent acetylation contributes to the selective regulation of genes involved in glucose metabolism of mammalian cells. It would be interesting to speculate on the regulatory effects of *acl12* overexpression on glycolysis and ultimately IA production.

Furthermore, this research showed the importance of aligning fermentation optimization with genetic engineering. While overexpression of *acl12* improved overall IA production in M12++ medium, the use of MRA medium showed that elevated levels of NH_4_NO_3_, magnesium and iron (III) already had significant impact on IA titers that could be reached by this new strain lineage. Subsequent efforts on fermentation optimization revealed the importance of NO_3_^−^ and NH4^+^ availability during IA production. However, extensive research on this topic suggests that IA production should remain at nitrogen limiting conditions, which would direct global gene expression towards overflow metabolism of nitrogen-free compounds such as IA [4, 42]. Therefore, nitrogen concentration has to be tightly regulated during the course of cultivation in order to ensure optimal IA production.

In our previous experiments, we have observed that CitB#99 and *acl12* overexpressing transformants start to degrade IA at titers between 15 and 20 g/L (Fig. 3) [45]. Interestingly, by maintaining NO_3_^−^ and NH_4_^+^ availability, both cessation of IA degradation and elevated titers could be established as shown by the cultivations in MRA medium as well as pulse-feeding experiments (Figs. 3, 4), suggesting a vital role of these compounds in IA detoxification. Literature describes the central role of these nitrogen sources in a specific pathway on which acid-tolerant microorganisms rely heavily to reduce the cytotoxic effects of weak acids in acidic environments [46, 47]. This pathway is referred to as the glutamate-dependent acid resistance mechanism (GDARM), in which NH_4_^+^ and/or NO_3_^−^ are essential precursors for generation of glutamate allowing a proton-consuming decarboxylation reaction into γ-aminobutyric acid (GABA) to ultimately increase the alkalinity of the cytoplasm [46, 48–52]. Kubicek et al. [53] found that *A. niger* accumulates GABA during acidogenesis, suggesting that *A.* *niger* also relies on this system to cope with acidic environments. Supported by our results, maintaining NH_4_^+^ or NO_3_^−^ availability, therefore, seems essential for *A. niger* to cope with high IA titers, continue IA production and prevent its degradation in response to weak-acid stress. Note that in this pathway, NO_3_^−^ first has to be converted into NH_4_^+^ to function as precursor for glutamate. This energy-draining process in the form of NADPH consumption causes an increase in glycolytic flux, in addition to the fact that ammonium salts further decrease the extracellular pH as they are consumed while nitrate does not, together might explain the bigger impact of NO_3_^−^ over NH_4_^+^ observed during the pulse-feed experiments. Note that in the shake flask pulse-feed experiments acidifying NH_4_^+^ sources have been used in the form of NH_4_Cl and NH_4_NO_3_; whereas in the controlled fed-batch cultivations NH_4_OH, which is not an acidifying ammonium source, has been used to maintain the extracellular pH at 3.0 and feed NH_4_^+^. The results presented here show that the fermentation protocol with the ACL G1 Z strain that was designed is robust, in the sense that applying these conditions in different fermentation labs and different equipment (see Additional file 1: Figure S6) resulted in similarly high IA titer, yield and productivity (56.6 g/L IA; 0.28 g IA/g glucose; 0.18 g IA/L/h, respectively). Moreover in this specific case, no CA was detected (Additional file 1: Figure S6) and the carbon balance accounted for 96.6% of total carbon that was added to the fermenter (Additional file 1: Table S3).

Simultaneously with the findings on NO_3_^−^/NH_4_^+^ availability in relation to IA degradation, we have observed upregulation of the IA bioconversion pathway in *A. niger* responsible for detoxifying the extracellular medium of IA during high producing conditions, resembling the IA degrading pathway of *A. terreus* described by Chen et al. [45, 54]. Knock-out of the key pathway-associated genes in CitB#99 resulted in cessation of IA bioconversion and elevated titers [45]. However, as these high IA titers have shown to result in stronger growth effects possibly also due to weak-acid stress, combining this knock-out of the IA degradation pathway in combination with NH_4_^+^/NO_3_^−^ balancing conditions is a clear target for ongoing research to improve IA production in *A. niger*.

## Conclusions

Ultimately, this study showed that the targeting of genes that improve glycolytic flux, more specifically the genes encoding ATP-citrate lyase *acl12*, constitutes a successful strategy for the improvement of IA production. This improvement manifests in increased IA titer, yield and productivity. Furthermore, this study also highlights the importance of medium composition and culture conditions for IA production. In shake flask cultivation, optimization of the cultivation conditions by supplementation of an alkalizing nitrogen source showed great potential for improvement of IA production in *A. niger* and these results were also shown in controlled fed-batch cultivations at 10-L scale. These strategies all yield great promise for future improvement of biological IA production to ensure its viability in the rising bio-based economy.

## Supplementary information

**Additional file 1: Figure S1.** Design of a*cl1* and *acl2* expression cassette. **Figure S2.** Shake flask cultivation of CitB#99 ACL G1 Z. **Table S1.** Relative copy numbers of introduced *acl12* and IA titers achieved in shake flask cultivations. **Figure S3.** Southern-Blot results of selected CitB#99-*acl* transformants. **Table S2.** Carbon balance of 10L fed-batch bioreactor cultivation of ACL G1 Z. **Figure S4.** Dissolved oxygen profile and pH profile during 10 L controlled fed-batch cultivation of strain CitB#99-ACL G1 Z. **Figure S5.** Total nitrogen concentration during 10 L controlled fed-batch cultivation of ACL G1 Z. **Figure S6.** Repeat fed-batch cultivation of ACL G1 Z performed in 5 L BioFlo 320 (Eppendorf) controlled bioreactors. **Table S3.** Carbon balance of repeat fed-batch 5 L bioreactor cultivation of ACL G1 Z.

## Data Availability

Transcriptome data will be uploaded on GEO.
