# Supplementary material for: Metabolic engineering with ATP-citrate lyase and nitrogen source supplementation improves itaconic acid production in Aspergillus niger
Source: Biotechnol Biofuels. 2019 Sep 30;12:233. doi: 10.1186/s13068-019-1577-6 (PMC6767652; doi:10.1186/s13068-019-1577-6)
Supplement: Supplementary file 1 — Additional file 1: Figure S1. Design of acl1 and acl2 expression cassette. Figure S2. Shake flask cultivation of CitB#99 ACL G1 Z. Table S1. Relative copy numbers of introduced acl12 and IA titers achieved in shake flask cultivations. Figure S3. Southern-Blot results of selected CitB#99-acl transformants. Table S2. Carbon balance of 10L fed-batch bioreactor cultivation of ACL G1 Z. Figure S4. Dissolved oxygen profile and pH profile during 10 L controlled fed-batch cultivation of strain CitB#99-ACL G1 Z. Figure S5. Total nitrogen concentration during 10 L controlled fed-batch cultivation of ACL G1 Z. Figure S6. Repeat fed-batch cultivation of ACL G1 Z performed in 5 L BioFlo 320 (Eppendorf) controlled bioreactors. Table S3. Carbon balance of repeat fed-batch 5 L bioreactor cultivation of ACL G1 Z. [file 13068_2019_1577_MOESM1_ESM.docx]

**Figure S1: Design of a*cl1* and *acl2* expression cassette.**


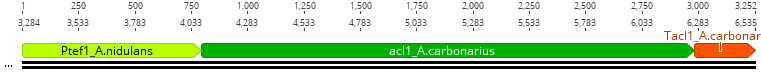


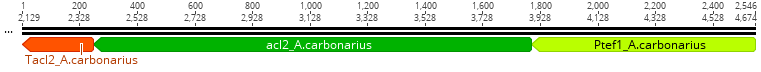


**Figure S2: Shake flask cultivation of CitB#99 ACL G1 Z.**


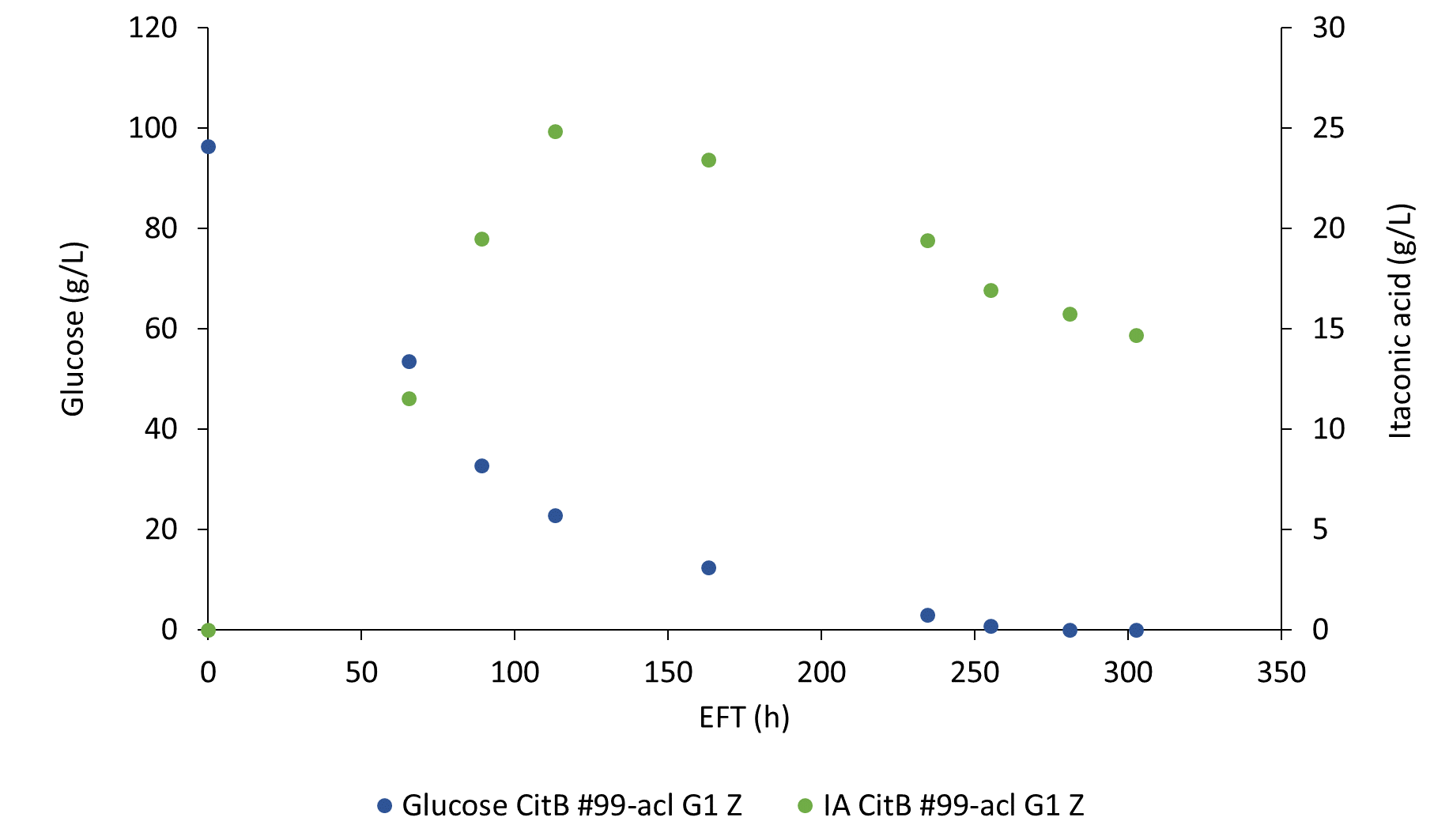


**Table S1: Relative copy numbers of introduced *acl12* and IA titers achieved in shake flask cultivations. Results are from a single representative experiment.**

| **Strain of *A. niger*** | **Max. IA titer (g/L)** | **Southern blot results** | |
| --- | --- | --- | --- |
|  |  | **(rel. gene copy no.)** | |
|  |  | ***acl1*** | ***acl2*** |
| **CitB#99** | 15.6 | 0 | 0 |
| **CitB#99-*acl* A9 G** | 17.9 | 2 | 4 |
| **CitB#99-*acl* B11 G** | 18.0 | 1 | 4-5 |
| **CitB#99-*acl* E11 Z** | 18.4 | 8-10 | 10 |
| **CitB#99-*acl* D9 R** | 18.4 | 3 | 2 |
| **CitB#99-*acl* G1 Z** | 20.6 | 1 | 3 |

**Figure S3: Southern-Blot results of selected CitB#99-*acl* transformants.**


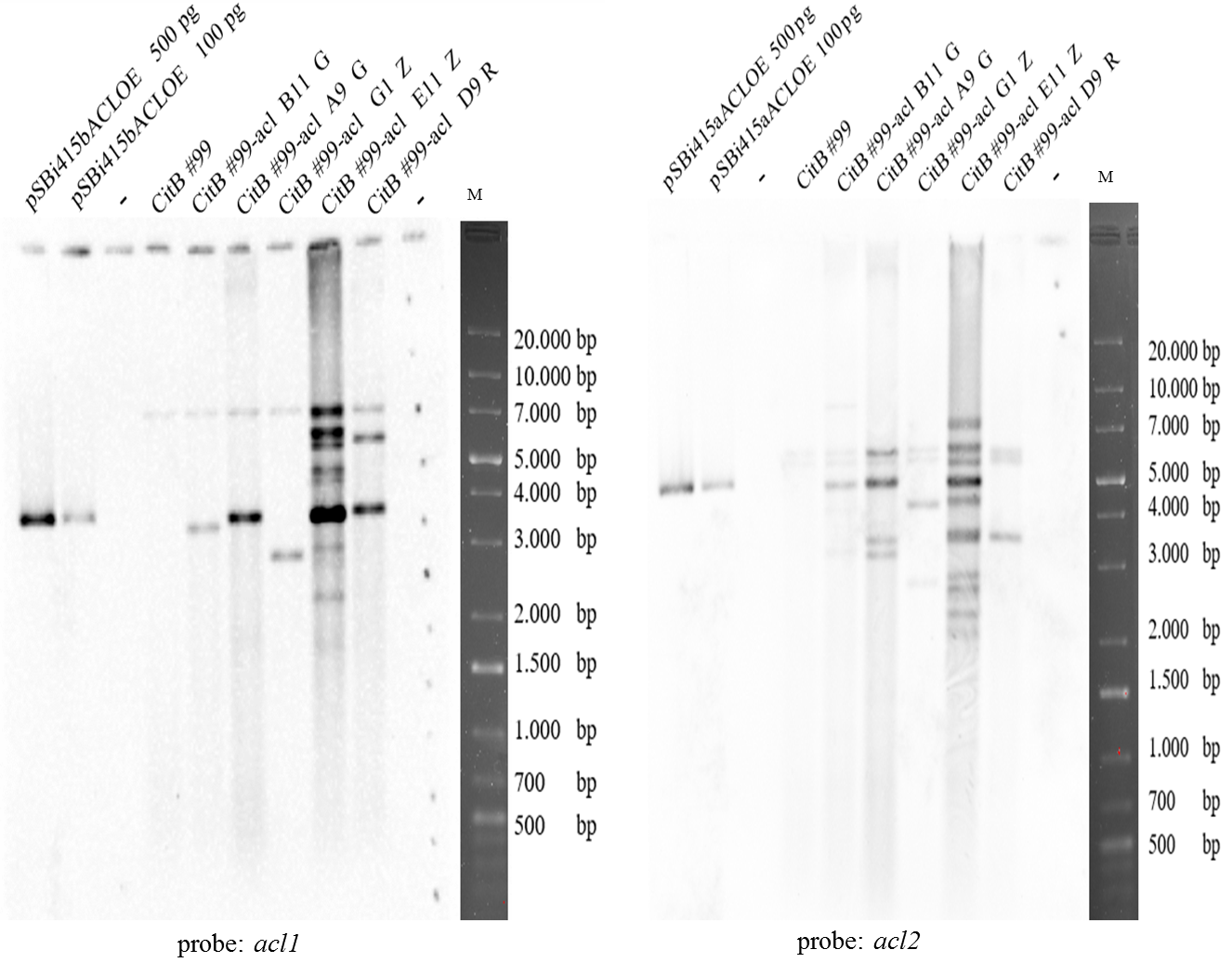


Note that the single fragment present for CitB#99 upon hybridization with the *acl1* probe corresponds to a hybridization event with the native *acl1* gene of *A. niger* and is excluded in the estimation of relative gene copy numbers for all CitB#99-*acl* transformants. Also note that the two fragments present for CitB#99 upon hybridization with the *acl2* probe correspond to a hybridization event with the native *acl2* gene of *A. niger* (on the lower fragment) and with the promoter region of the *tef1* gene of *A. niger* (on the upper fragment).

**Table S2:** **Carbon balance of 10L fed-batch bioreactor cultivation of ACL G1 Z. IA: itaconic acid; CA: citric acid.**

| **mmol C/fermentor (percentage of the total)** | | | | | | |
| --- | --- | --- | --- | --- | --- | --- |
| **Total C added** | **CO_2_ produced** | **IA produced** | **CA produced** | **Biomass produced** | **Remaining glucose** | **Others** |
| 71936 (100%) | 22300 (31%) | 19209 (26,7%) | 3546 (4,9%) | 7849 (10,9%) | 6888 (9,7%) | 4848(6,7%) |

**Figure S4: (A) pH profile during fed-batch cultivation of strain CitB#99- ACL G1 Z. (B) Dissolved oxygen (DO) profile during fed-batch cultivation.**


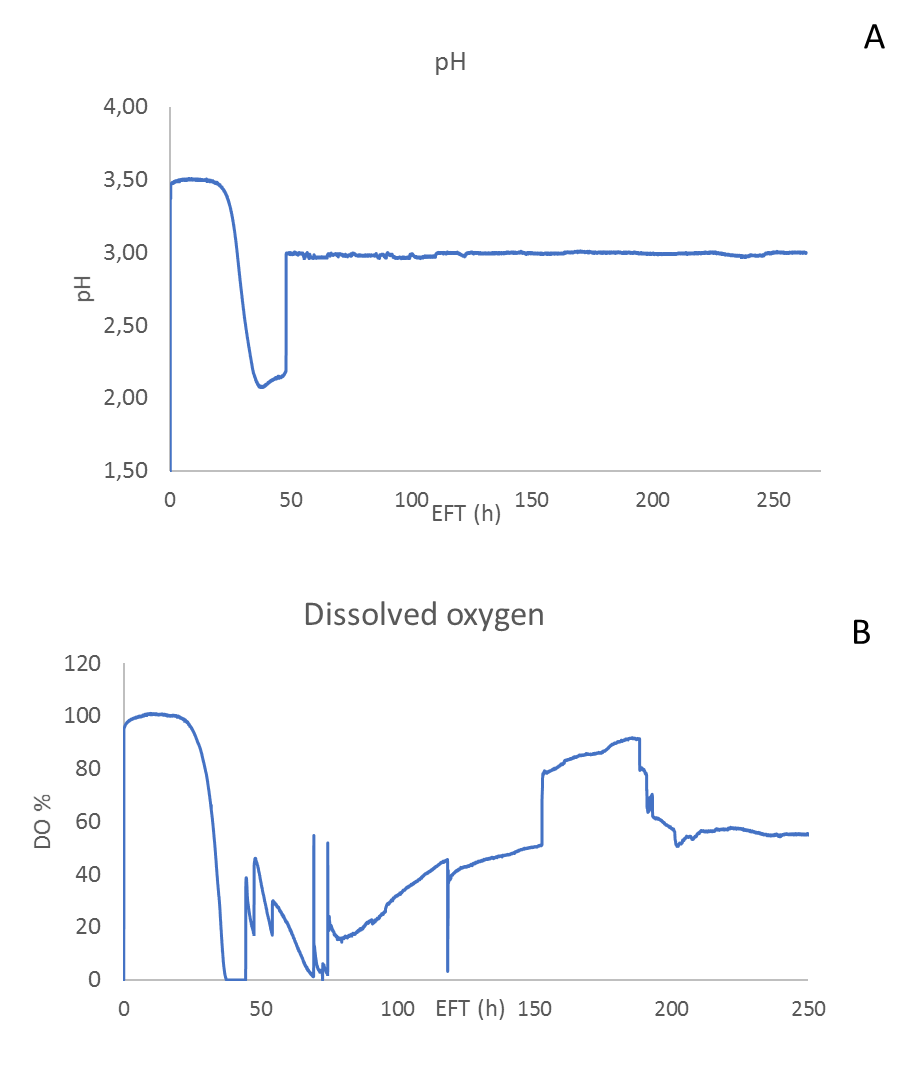


**Figure S5:** **Total nitrogen concentration during 10L controlled fed-batch cultivation of ACL G1 Z.**


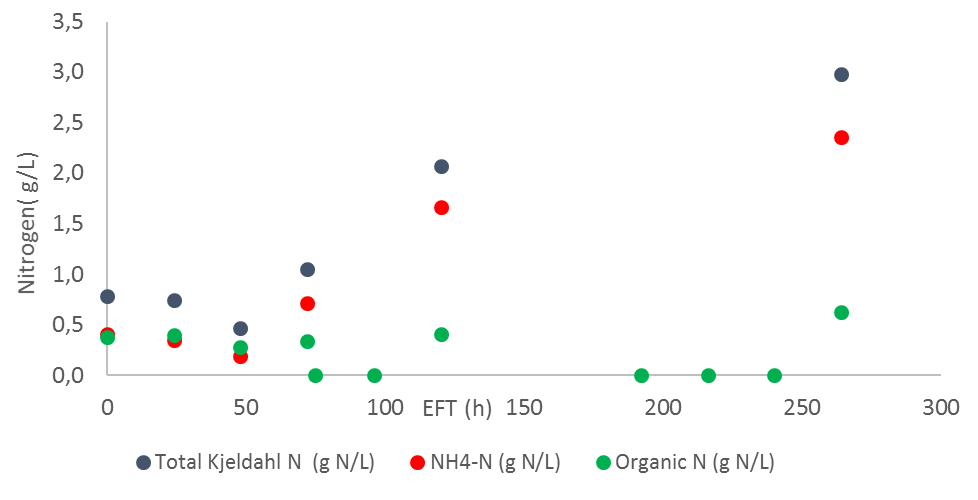


**Figure S6:** **Repeat fed-batch cultivation of ACL G1 Z performed in 5L BioFlo 320 (Eppendorf) controlled bioreactors. (A) Glucose consumption and IA production of CitB#99 ACL G1 Z. (B) pH profile during repeat fed-batch cultivation. (C) DO profile during repeat fed-batch cultivation.**


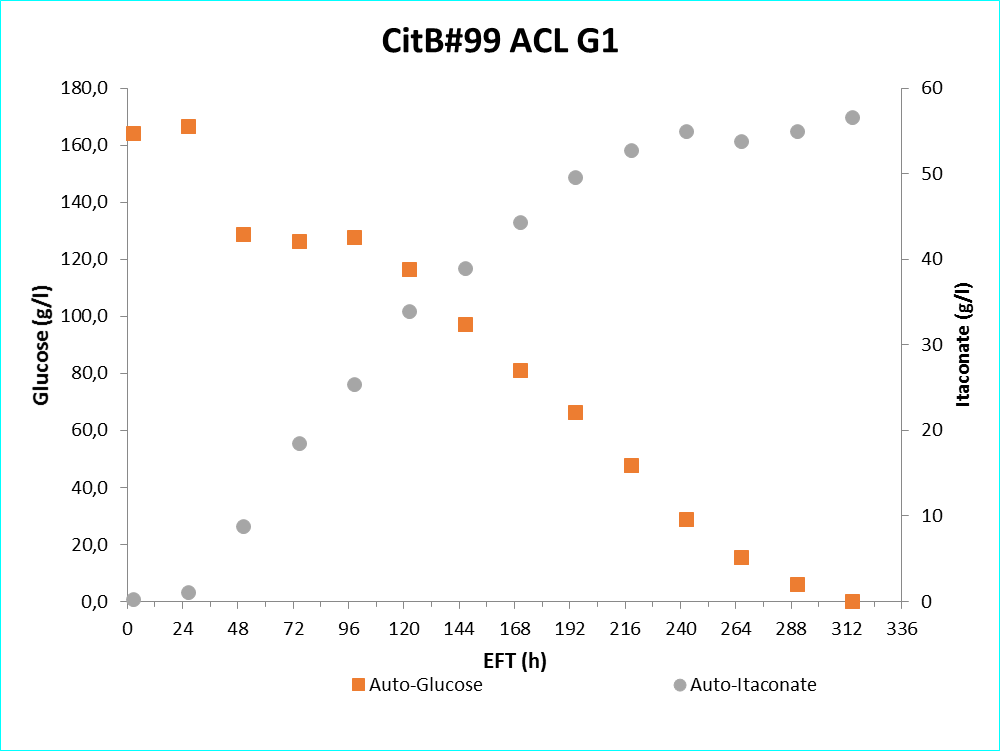


**B**

**C**

**Table S3:** **Carbon balance of repeat fed-batch 5L bioreactor cultivation of ACL G1 Z. IA= Itaconic acid.**

| **mmol C/fermentor (percentage of the total)** | | | | |
| --- | --- | --- | --- | --- |
| **Total C added** | **CO_2_ produced** | **IA produced** | **Biomass produced** | **Others** |
| 33737 (100%) | 13183 (39,1%) | 10878 (32,2%) | 4585 (13,6%) | 3965 (11,8%) |
